# Spatially Resolved Activity-based Proteomic Profiles of the Murine Small Intestinal Lipases

**DOI:** 10.1074/mcp.RA120.002171

**Published:** 2020-10-06

**Authors:** Matthias Schittmayer, Nemanja Vujic, Barbara Darnhofer, Melanie Korbelius, Sophie Honeder, Dagmar Kratky, Ruth Birner-Gruenberger

**Affiliations:** 1 Institute of Chemical Technologies and Analytics, Vienna University of Technology, Vienna, Austria; 2 Diagnostic and Research Institute of Pathology, Medical University Graz, Graz, Austria; 3 Gottfried Schatz Research Center, Medical University Graz, Graz, Austria; 4 BioTechMed-Graz, Graz, Austria

**Keywords:** lipid droplet, label-free quantification, mouse models, mass spectrometry, mechanism of action, molecular probes, activity based proteomics, small intestine

## Abstract

Despite the crucial function of the small intestine in nutrient uptake our understanding of the molecular events underlying the digestive function is still rudimentary. Recent studies demonstrated that enterocytes do not direct the entire dietary triacylglycerol toward immediate chylomicron synthesis. Especially after high-fat challenges, parts of the resynthesized triacylglycerol are packaged into cytosolic lipid droplets for transient storage in the endothelial layer of the small intestine. The reason for this temporary storage of triacylglycerol is not completely understood. To utilize lipids from cytosolic lipid droplets for chylomicron synthesis in the endoplasmic reticulum, stored triacylglycerol has to be hydrolyzed either by cytosolic lipolysis or lipophagy. Interestingly, triacylglycerol storage and chylomicron secretion rates are unevenly distributed along the small intestine, with the proximal jejunum exhibiting the highest intermittent storage capacity. We hypothesize that correlating hydrolytic enzyme activities with the reported distribution of triacylglycerol storage and chylomicron secretion in different sections of the small intestine is a promising strategy to determine key enzymes in triacylglycerol remobilization. We employed a serine hydrolase specific activity-based labeling approach in combination with quantitative proteomics to identify and rank hydrolases based on their relative activity in 11 sections of the small intestine. Moreover, we identified several clusters of enzymes showing similar activity distribution along the small intestine. Merging our activity-based results with substrate specificity and subcellular localization known from previous studies, carboxylesterase 2e and arylacetamide deacetylase emerge as promising candidates for triacylglycerol mobilization from cytosolic lipid droplets in enterocytes.

## Abstract (precis)

Remobilization of temporary lipid stores in the small intestine is an incompletely understood process but it is evident that lipids are transported onwards to the liver and peripheral tissues by chylomicrons. This study correlates lipase activity profiles with inter-prandial chylomicron secretion profiles to pinpoint the key enzymes responsible for remobilization of lipids in the small intestine.

## Graphical Abstract

**Figure:**
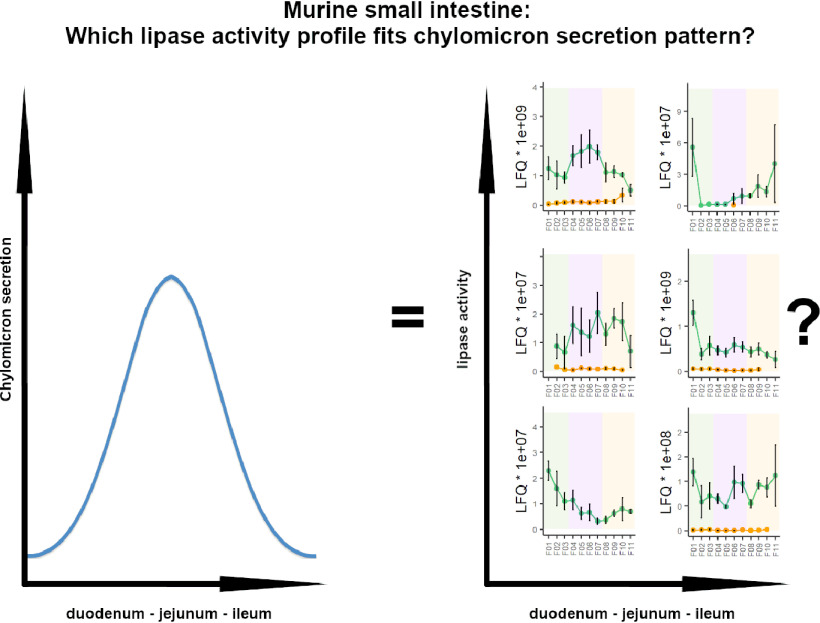

**Highlights**

- Activity based serine hydrolase profiles in 11 fractions along the murine small intestine.
- AADAC and Ces2e activity profiles correlate with chylomicron secretion.
- Ces2e shows the highest activity based enrichment in the entire small intestine.

The main role of the digestive system is the breakdown of nutrients into absorbable building blocks which are subsequently taken up by the small intestine and distributed in the body. Lipids are a major dietary energy and carbon source and the most energy dense molecules utilized by the organism. Their energy content is twice as high as compared with proteins and carbohydrates. Because of their high nutritive value, the uptake of dietary fat in the small intestine is a highly efficient process with more than 95% of lipids being absorbed by the intestinal mucosa (1). To pass the endothelial barrier, dietary lipids which mainly consist of triacylglycerol (TAG) must be broken down into free fatty acids (FFA) and monoacylglycerol (MAG) in the intestinal lumen. Lipid digestion starts directly after ingestion by the action of acidic lipases, namely lingual lipase (2) and gastric lipase, and is continued in the most proximal part of the small intestine, the duodenum (3). Here, the acidic gastric content is rapidly neutralized by bile salts and bicarbonate secretions of the intestinal mucosa (4) and pancreatic lipases active at neutral pH complete the hydrolysis in the following section of the small intestine, the jejunum (Fig. 1*A*). The resulting FFA and MAG are emulsified with the help of phospholipids and bile acids to form mixed micelles which are then taken up by the enterocytes lining the villi of the small intestine (5). Within the endoplasmic reticulum (ER) lumen of enterocytes, FFA and MAG are re-esterified to TAG which are then used to lipidate nascent chylomicrons (CM). Subsequently, CM are distributed via the lymphatic system and blood stream to the whole body (6) (Fig. 1*B*). More recently, it has become evident that not all TAG absorbed by enterocytes is immediately secreted in the form of CM. Particularly after a dietary fat challenge, part of the re-synthesized TAG is transiently stored in the form of cytosolic lipid droplets (CLD) (7). The reasons for this intermediate storage of TAG are currently not completely understood. Alleviating lipotoxicity to enterocytes (1), limits in CM assembly rate (8) and smoothing of the post-prandial peak of blood hypertriglyceridemia (9) have been suggested as potential mechanism underlying the intermediate storage of TAG in CLD. Rather than being evenly distributed across the small intestine, the proximal jejunum exhibits the highest TAG storage and CM secretion (1), indicating distinct functions of the small intestine sections in the process of lipid assimilation. Once stored in CLD, lipids are either remobilized in the inter-prandial phase to sustain systemic energy demand or by various stimuli that announce demand for capacity of renewed CLD storage, *e.g.* a sequential meal containing either lipids or glucose (10). In the latter case, a CM peak arises in plasma well before lipids from the second meal have been digested in the intestinal lumen. Therefore, these CM must be synthesized from CLD derived lipids. As intact TAG cannot pass cellular membranes, CLD derived TAG must be hydrolyzed to FFA and MAG for transport across the ER membrane. TAG hydrolysis can either take place in the cytosol (7) or in lipophagosomes (11). Although the latter mechanism has been suggested to degrade a subset of newly synthesized CLD under conditions of nutritional stress (11), cytosolic lipolysis in enterocytes is incompletely understood. Mice lacking adipose triglyceride lipase (ATGL, Q8BJ56, *Pnpla2*) activity solely in the small intestine have increased enterocyte TAG content but no significant change of plasma TAG, cholesterol or nonesterified fatty acids was observed (12), indicating that CM assembly is not ATGL dependent. Likewise, targeted disruption of hormone sensitive lipase (HSL, P54310, *Lipe*) expression in murine enterocytes (13) only influences plasma cholesterol but not plasma TAG or enterocyte TAG levels. A recent study utilizing intestine specific double knock-out mice lacking both, ATGL and its activator alpha/beta hydrolase domain-containing protein 5 (ABHD5 alias CGI-58, Q9DBL9, *Abhd5*), showed prominent accumulation of TAG in the proximal parts of the small intestine (14). However, the same study revealed that this TAG originates from the basolateral side of the enterocyte and not from dietary lipids. In contrast, loss of carboxylesterase 1d (Ces1d, Q8VCT4, *Ces1d*), an ER resident lipase playing a major role in very low density lipoprotein (VLDL) assembly in the liver, causes significant reduction of CM secretion in *Ces1d*^−/−^/*Ldlr*^−/−^ mice (15). However, intestinal expression of Ces1d is among the lowest of the whole family of carboxylesterases (16) and other members of the family have been reported to influence CM secretion (Ces1g, Q8VCC2, *Ces1g*) (17), CM size (Ces2c, Q91WG0, *Ces2c*) (18) and fat absorption (Ces1g) (19) as well. For a list of all protein names, their abbreviations and corresponding gene names used in this study see supplemental Table S1.

**Fig. 1.**
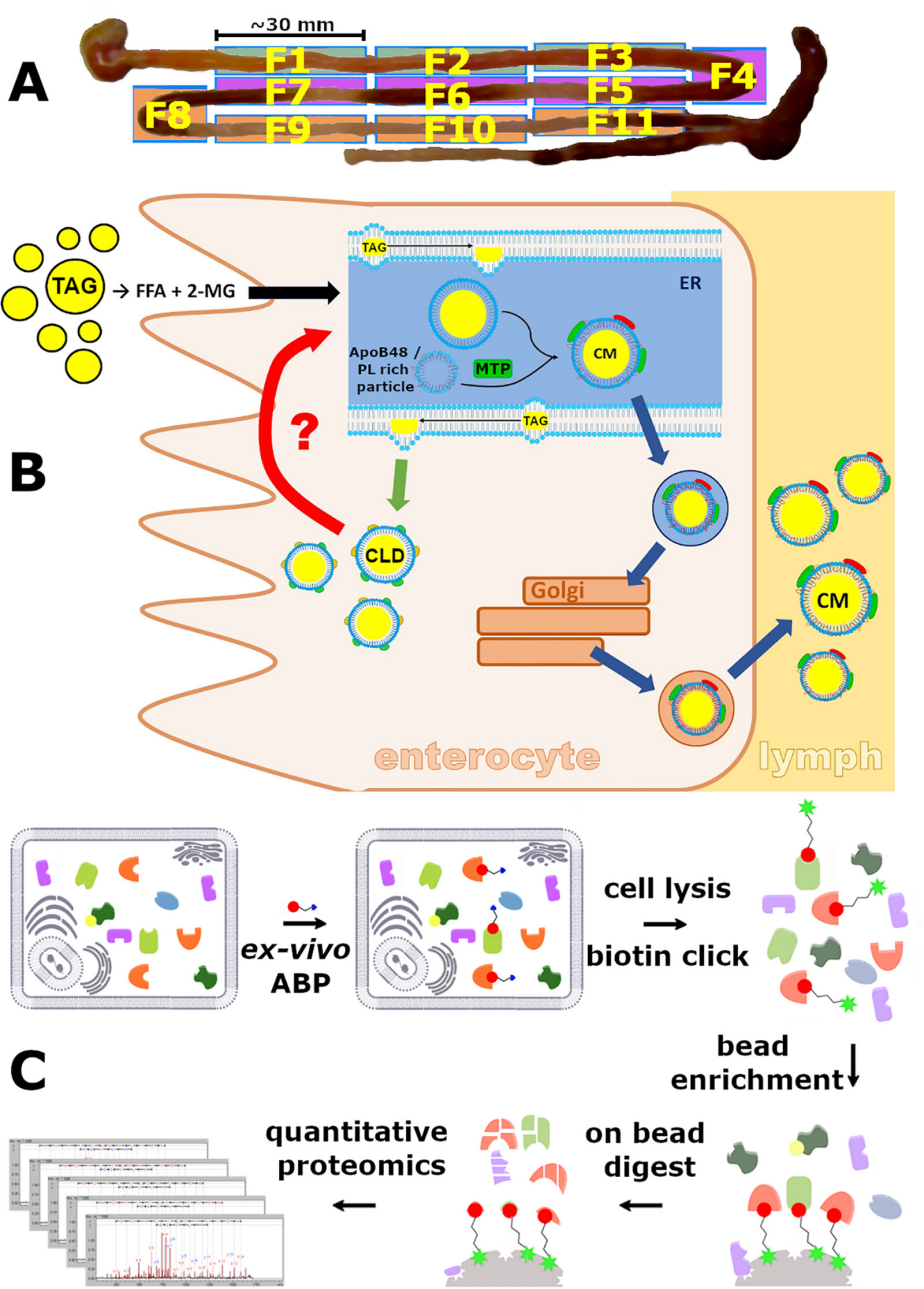
***Ex vivo* activity-based proteomics labeling of mouse small-intestine sections.** *A*, Fractions (F1-F11) of the small intestine. Green: duodenum (F1-F3), purple: jejunum (F4-F7), orange: ileum (F8-F11). *B*, Schematic of the remobilization process of cytosolic lipid droplets. *C*, Activity-based proteomics workflow; intact tissues are labeled with a cell permeable activity-based probe specific for serine hydrolases. TAG: triacylglycerol; ER: endoplasmic reticulum; CLD: cytosolic lipid droplet; FFA: free fatty acid; 2-MG: 2-monoacylglycerol; CM: chylomicron; Red question mark/arrow: function of unknown enzyme.

A major challenge in identifying enzymes involved in CLD remobilization in the small intestine is the multitude of esterases and other serine hydrolases expressed in enterocytes. To depict the enzymatic activities of the entire serine hydrolase class we employed a technology termed activity-based proteomics (20–23). Activity-based probes (ABP) are small molecule inhibitors that contain a reactive group specifically targeting a subset of enzymes on a mechanistic basis. Next to the reactive group and structural features mimicking natural substrates, ABP typically carry an analytical handle which allows subsequent detection or enrichment of labeled enzymes. This can either be a fluorophore, affinity tag or a tag for biorthogonal reactions (24, 25). As the binding of the probe is based on a common catalytic mechanism, only enzymatically active proteins are labeled, which yields a biologically highly meaningful readout not reflected by mere protein abundance.

In this study, we aimed to identify and rank hydrolases participating in intestinal lipid metabolism by activity-based proteomics. We hypothesize that correlating enzymatic activities of hydrolases with the reported distribution of TAG storage and CM secretion in different sections of the small intestine is a promising strategy to determine key players in TAG remobilization. To sort hydrolases based on their relative activity in the different sections of the small intestine we harnessed a serine hydrolase specific (26) activity-based strategy to label active hydrolases in freshly harvested enterocytes (Fig. 1*C*). By employing (1) enrichment over nonprobed control samples and (2) abundance of enriched hydrolases from individual small intestine sections we identified the most active lipases in 11 fractions of murine small intestine.

## MATERIALS AND METHODS

### 

#### 

##### Reagents

All reagents were purchased from Sigma-Aldrich, Vienna, Austria, if not stated otherwise.

##### Activity-Based Probe Synthesis

ABP C6 (supplemental Fig. S1) was synthesized according to previously published protocols (27). In short, (3-Azidopropyl)phosphonic acid was synthesized by SN2 reaction from (3-Bromopropyl)phosphonic acid and sodium azide. 1-Hexanol was coupled to phosponic acid employing EDC.HCl and the product was converted to the fluorophosphonate ester employing (diethylamino)sulfur trifluoride. Subsequently, the product was converted to the p-Nitrophenol phosphonate in dry dichloromethane with 0.1 Eq tetrazole as catalyst. Product identity was confirmed by 1H-NMR ((CDCl_3_): δ 8.19 (d, 2 H), δ 7.31 (d, 2 H), 4.06 (m, 2 H), 3.37 (t, 2 H), 1.93 (m, 4 H), 1.55 (m, 4 H), 1.21 (m, 4 H), 0.81 (t, 3 H)).

##### Animals and Tissue Isolation

Eleven-week old male C57BL/6J mice were fed with Western-type diet (TD88137 mod., 21% fat, 0.2% cholesterol; Ssniff Spezialdiaeten GmbH, Soest, Germany) for 4 weeks. Thereafter, animals were fasted overnight (12 h) and gavaged with 120 µl of corn oil. Two hours post-gavage, mice were sacrificed by cervical dislocation, small intestines were dissected, briefly washed in PBS and cut in ∼ 3 cm long pieces. Luminal sides of each piece were scraped, scrapings were thoroughly mixed, split in 2 parts and used as stated below. The first 3 fractions were designated as duodenum, fractions 4-7 as jejunum, and the 4 most distal fractions as ileum.

##### Activity Based Labeling and Cell Lysis

Each part of the scraped tissue was collected in Eppendorf tubes containing 400 µL PBS in the absence or presence of ABP C6 (Hexyl 4-nitrophenyl (3-azidopropyl)phosphonate, 30 μm), vortexed vigorously and immediately incubated at 37 °C under shaking (500 rpm) for 2 h. During this incubation time, the C6 probe specifically labeled active serine hydrolases within their native environment, *i.e.* the intact enterocytes. Thereafter, cell scrapings were lysed by sonication (10 s) and the cell debris was removed by centrifugation at 7000 × *g* for 10 min at 4 °C. The protein concentration was determined by BCA assay (Thermo Scientific, Vienna, Austria) and 500 µg protein or the whole fractions (where total protein amount was less than 500 µg) was subjected to acetone precipitation. Re-solubilization, reduction and alkylation were performed in 100 µL 4 M Urea, 2% SDS, 0.25 M NaCl containing 20 mm TCEP and 60 mm NEM at 95 °C for 10 min.

##### Biotin Click Reaction, Bead Enrichment and on Bead Digest

After addition of 900 pmol DBCO-TEV-Biotin strain promoted click linker (supplemental Fig. S2, PiChem, Austria), the click reaction was carried out under shaking at 37 °C and 500 rpm, overnight. Samples were diluted to SDS concentration < 0.1% and excess linker was removed employing 3 kDa cutoff filters. Retentate was washed twice with 500 µL 8 M urea, 100 mm TrisHCl, pH = 8.5 and once with 2 M urea, 100 mm TrisHCl pH = 8.5. Enrichment was performed using 12 µL streptavidin-agarose resin (Thermo Scientific) on an overhead rotator in plugged spin columns (Thermo Scientific) for 4 h. The beads were washed twice with 2 M urea, 100 mm TrisHCl pH = 8.5 before incubation with 200 µL 100 mm TrisHCl pH = 8.5 containing 100 ng trypsin for 10 h.

**Fig. 2.**
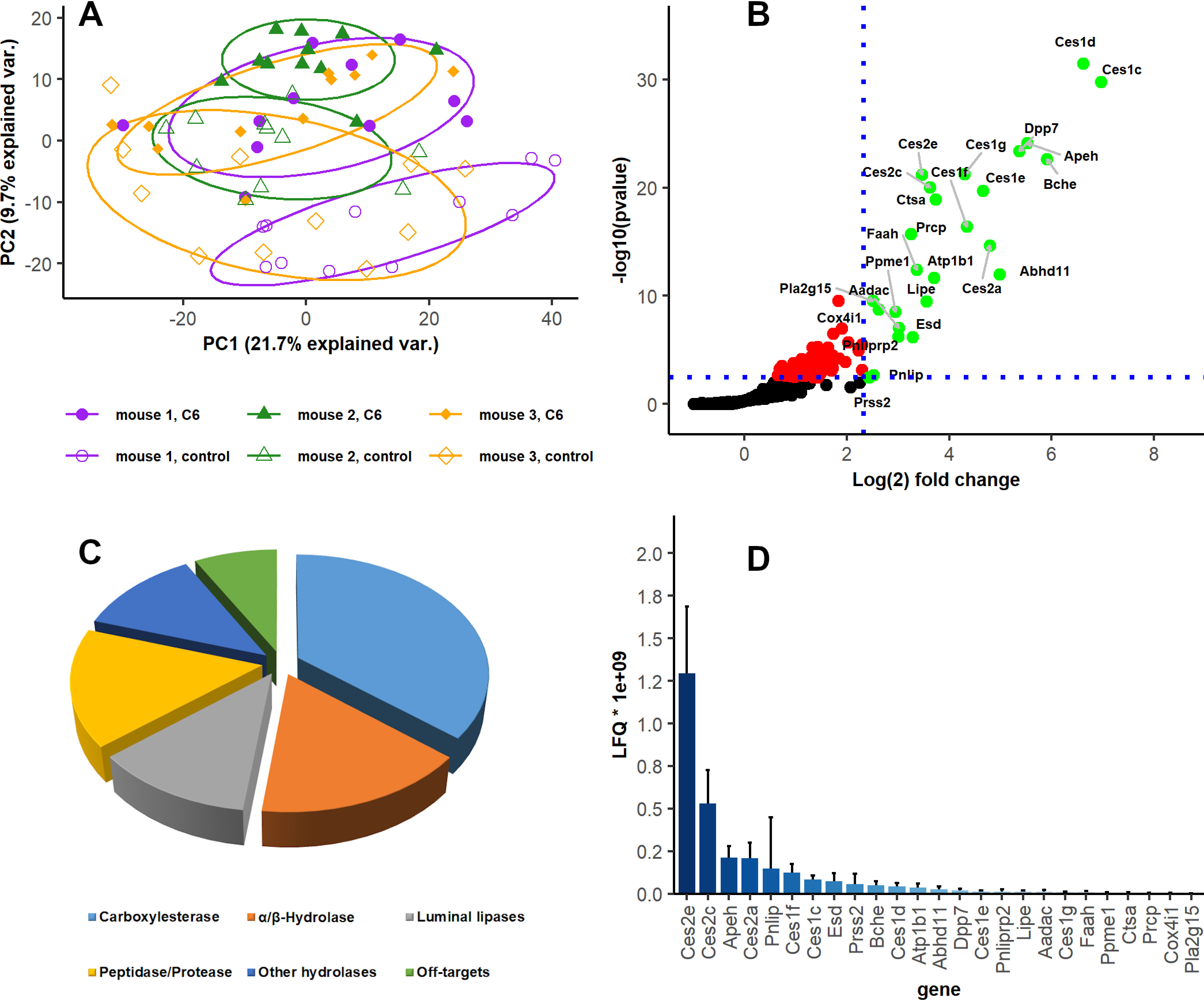
**ABP based enrichment of serine hydrolases in the murine small intestine.** *A*, Principal component analysis of the small intestine ABP data set. Mouse 1: purple circles; mouse 2: green triangle; mouse 3: orange diamond; probed samples: filled shapes, nonprobed controls: empty shapes. *B*, One-sided volcano plot of enzymes significantly enriched in probed samples *versus* Nonprobed controls in the entire small intestine (gene names); Student's *t* test; one sided; permutation-based FDR = 0.05 (31), minimum mean fold change = 5. *C*, Distribution of significantly enriched enzymes in individual serine hydrolase families. *D*, Hydrolases ranked by abundance (LFQ) in probed samples after activity-based enrichment.

##### LC–MS Conditions

The supernatant of the on-bead digest was harvested by centrifugation (500 × *g*, 5 min). Supernatants were brought to a volume of 100 μl with 1% trifluoroacetic acid in H_2_O and loaded onto self-packed 2-layer (18 gauge) 200 μl SDB-RPS Stage Tips (Empore SPE Disks, Sigma-Aldrich #66886-U). The samples were passed through the stage tips by centrifugation (5 min, 1500 × g, RT), washed with 0.2% trifluoroacetic acid, eluted with 5% NH_4_OH/80% acetonitrile into a new tube and dried using a vacuum concentrator. One fourth of each sample (re-dissolved in 2% acetonitrile/0.1% formic acid) was separated by nano-HPLC (Dionex Ultimate 3000) equipped with an Aurora Series Emitter nanocolumn (C18, 1.6 μm, 120 Å, 250 × 0.075 mm, IonOpticks). Separation was carried out at 50 °C at a flow rate of 300 nL/min using the following gradient (A: 0.1% formic acid in water, B: 0.1% formic acid in acetonitrile): 0–18 min: 2% B; 18–100 min: 2-25% B; 100–107 min: 25–35% B; 107–108 min: 35–95% B; 108–118 min: 95% B; 118–118 min: 95–2% B; 118–133 min: 2% B. Samples were analyzed using a Thermo Orbitrap Velos Pro in positive ion mode by alternating full scan MS (m/z 300 to 2000; 60,000 resolution) in the ICR cell and MS/MS by CID/Top10 in the ion trap with dynamic exclusion enabled.

##### Experimental Design and Statistical Rationale

The small intestine of 3 mice (3 biological replicates) was used for this study. Each intestine was cut into 11 longitudinal fractions. Enterocytes scraped from each of the fractions were split into two parts for ABP experiment and nonprobed control. A total of 66 (3 × 11 × 2) samples were employed for label free quantification (LFQ) and statistical analysis (see subsection Data analysis).

##### Data Analysis

Data were analyzed using MaxQuant 1.6.6.0 (28) searching Uniprot *Mus musculus* database (downloaded on Apr 28, 2019; 17013 entries) plus cRAP database (Mar 4, 2019); decoy database was reverted. The following search settings were used: N-Ethylmaleimide was set as fixed modification (C), oxidation (M) and acetylation (K) were considered as variable modifications. Enzyme trypsin, max. missed cleavage sites: 2; acceptance parameters for identification: 1% PSM FDR; 1% protein FDR and 1% site decoy fraction. LFQ was done using the match between runs feature (0.7 min match window), minimum of 2 ratio counts of quantified razor and unique peptides. First search tolerances were presets for Bruker Q-TOF (20 ppm peptide tolerance for first search, 10 ppm for main search). Data processing was performed with Perseus software version 1.6.6.0 (29). Data were filtered for decoy hits, contaminants and proteins only identified by modified peptides. After log2 transformation, proteins were filtered for containing at least 2 valid values in probed or control groups. Missing intensities were imputed with random values taken from the Gaussian distribution of values using default parameters (width of 0.3 and downshift of 1.8). Identified proteins were annotated with Pfam using the annotation tool integrated in Perseus.

Statistical analysis of proteins in probed *versus* Nonprobed samples was performed employing a one-sided Student's *t* test. Multi-testing correction was either done in Perseus based on significance analysis of microarrays (30) with parameters S0 = 2 and FDR = 0.05 or in RStudio (1.1.456) based on permutation based FDR using the qvalue package (31) (FDR = 0.05). The same strategy and parameters were employed to detect changes of hydrolase activities between individual fractions and sections of the small intestine but employing a two-sided Student's *t* test in this case.

The MS proteomics data were deposited to the ProteomeXchange Consortium via the PRIDE (32) partner repository with the data set identifier PXD019593 and doi:10.6019/PXD019593. For single peptide identification review identified spectra were uploaded to MS-Viewer (key: yxiu2pog1a)

## RESULTS

### 

#### 

##### An Overview of Lipid Hydrolases Active in the Murine Small Intestine

To provide an overview of identified proteins and enriched hydrolases, each of the 11 small intestine sections per mouse were treated as fractions of one sample. After correcting for potential contaminants and proteins only represented by modified peptides, this approach yielded 2184 proteins (at 1% peptide FDR) from 66 individual samples (3mice, 11 small intestinal fractions, each probed and nonprobed). Filtering for at least two valid values in at least one group reduced the number of proteins to 1415, whereas filtering for two identifications per fraction yielded 1307 proteins (supplemental Table. S2). Principal component analysis (PCA) of protein abundance revealed partial, but not complete separation of probed and nonprobed samples and an expected large variation between individual mice (Fig. 2*A*).

The 1307 proteins identified in individual fractions were spread over many enzyme classes and most were shared between probed and nonprobed samples, indicating unspecific binding of unlabeled proteins to streptavidin beads. Indeed, stringent statistical testing (permutation based FDR < 0.05, minimum fold change = 5) revealed that only 25 of the 1307 fraction specific proteins (supplemental Table S2) were significantly enriched in the probed as compared with nonprobed samples (Fig. 2*B*). Within these 25 significantly enriched enzymes, 22 belonged to the protein class of hydrolases (EC 3.X.X.X). One of the 3 remaining significantly enriched proteins was group XV phospholipase A2 (LPLA2, Q8VEB4, *Pla2g15*), an enzyme with reported acyltransferase and phospholipase activity classified as EC 2.X.X.X, despite hydrolase activity. The two remaining proteins were an isoform of cytochrome *c* oxidase subunit 4 (COX IV-1, P19783, *Cox4i1*) and a Na^+^,K^+^-transporting ATPase (ATP1B1, P14094, *Atp1b1*) which was reported to tightly bind to phospholipids (33). Nevertheless, these two proteins were considered as off-targets, yielding an enzyme class specific enrichment of 92% for the highly confident hits. As annotated by protein families (Pfam), the largest group of enriched hydrolases belonged to the group of carboxylesterases (9 members), followed by the alpha/beta hydrolase superfamily (4 members) (Fig. 2*C*). Out of the >200 total serine hydrolases described in literature we identified 32 in the complete data set. Despite the stringent filtering applied (> 5-fold enrichment), 23 out of the 32 serine hydrolases (72%) were significantly enriched (also counting LPLA2 which does have an active site serine).

Among the significantly enriched enzymes, we identified lipid hydrolases from the intestinal lumen as well as from the interior of enterocytes. The luminal enzymes included pancreatic triacylglycerol lipase (PL, Q6P8U6, *Pnlip*), pancreatic lipase-related protein 2 (PL-RP2, P17892, *Pnliprp2*) but also other known secreted hydrolases such as cholinesterase (CHLE, Q03311, *Bche*) and dipeptidyl peptidase 7 (DPP7, Q9ET22, *Dpp7*).

Regarding the intracellular lipid hydrolases, we successfully enriched HSL, alpha/beta hydrolase-domain containing protein 11 (ABHD11, Q8NFV4, *Abhd11*), arylacetamide deacetylase (AADAC, Q99PG0, *Aadac*) and several members of the carboxylesterase family (Ces) employing data of the entire small intestine. Among Ces, members of the Ces subfamily 1 reached the highest confidence p-values for enrichment (Fig. 2*B*) but were overall less abundant in the enriched fractions than those of Ces subfamily 2 as determined by LFQ (Fig. 2*D*). The detected LFQ values are partially in line with mRNA-based expression data (16), with Ces2 variants being the most abundant subfamily in the entire small intestine.

##### Enriched Lipid Hydrolases in Duodenum, Jejunum and Ileum

**Fig. 3.**
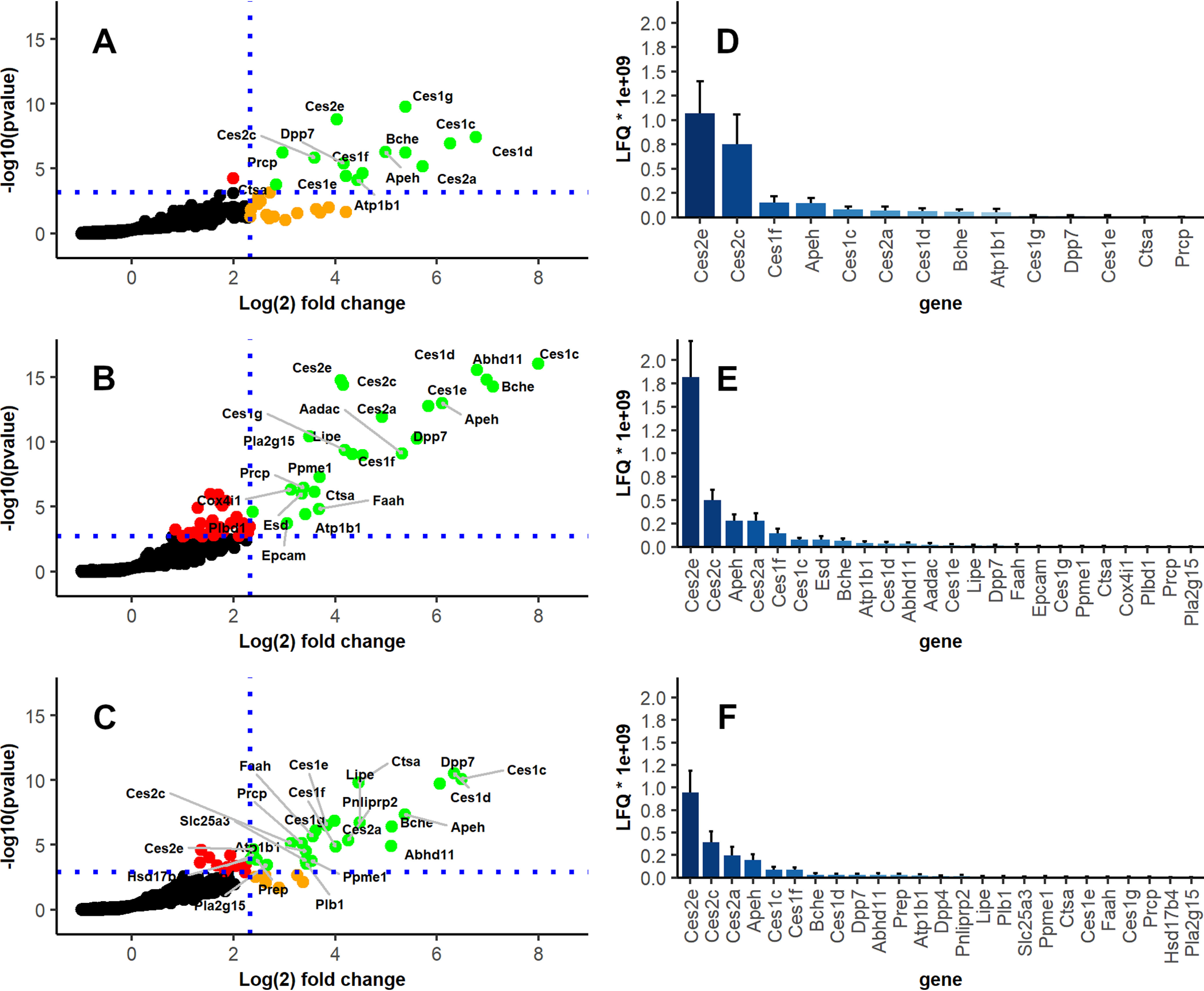
**Enriched lipid hydrolases in murine duodenal, jejunal and ileal sections.** Enriched lipases (labeled with gene names) in (*A*) the duodenum, (*B*) jejunum and (*C*) ileum. Red dots did not pass fold change threshold (> 5-fold); orange dots did not pass statistical multi-testing threshold (q < 0.05); green dots exceed both fold change and q-value threshold; black: all others. Distribution of enriched enzyme abundance as judged by label free quantification (LFQ) in (*D*) duodenum, (*E*) jejunum and (*F*) ileum.

Limiting statistical testing exclusively to the duodenal fractions (fractions 1-3) resulted in 14 significantly enriched proteins (supplemental Table S3). In this most proximal part of the small intestine numerous members of the Ces1 and Ces2 subfamilies were significantly enriched (Fig. 3*A*). Ces1g reached the smallest p-value for ABP based enrichment, followed by Ces2e and Ces1d. Ces1d also showed the highest relative enrichment compared with nonprobed controls with a mean fold change > 100, whereas the most abundant duodenal enzyme with respect to absolute LFQ values was Ces2e, followed by Ces2c, Ces1f and the peptidase acylamino acid releasing enzyme (APH, Q8R146, *Apeh*, Fig. 3*D*). Between the three individual fractions of the duodenum, no esterase was significantly enriched after statistical multi-testing and 5-fold change filtering (supplemental Fig. S3). A marked increase in the number of significantly enriched esterases was detected in fraction 4 as compared with the three previous fractions and therefore fraction 4 was designated as the first jejunal fraction (supplemental Table S4). In addition to the Ces family members already identified in Duodenum, HSL, fatty -acid amide hydrolase 1(FAAH1, O08914, *Faah*), AADAC, ABHD11 and LPLA2 lipases were significantly enriched in jejunum (Fig. 3*B*). Ces1c was the most significantly enriched enzyme and showed the highest fold-change as compared with nonprobed jejunum fractions. The ileum is not physically delimited from the jejunum but is anatomically defined as having a smaller inner diameter, thinner walls and less circular folds (34). A second marked change in the enrichment of many hydrolases was observed in fraction 8. We defined this fraction as the beginning of the ileum, which correlates roughly to the most distal third of the small intestine. However, the number of significantly enriched lipases did not change considerably between jejunum (24) (supplemental Table S4) and ileum (25) (supplemental Table S5) and the canonical lipases HSL, FAAH, and ABHD11 were enriched in both distal sections of the small intestine (Fig. 3*B*, 3*C*). As in both other sections, Ces2e was the most abundant enriched enzyme (Fig. 3*F*).

##### Longitudinal Activity-Based Enrichment Profiles of Lipid Hydrolases

**Fig. 4.**
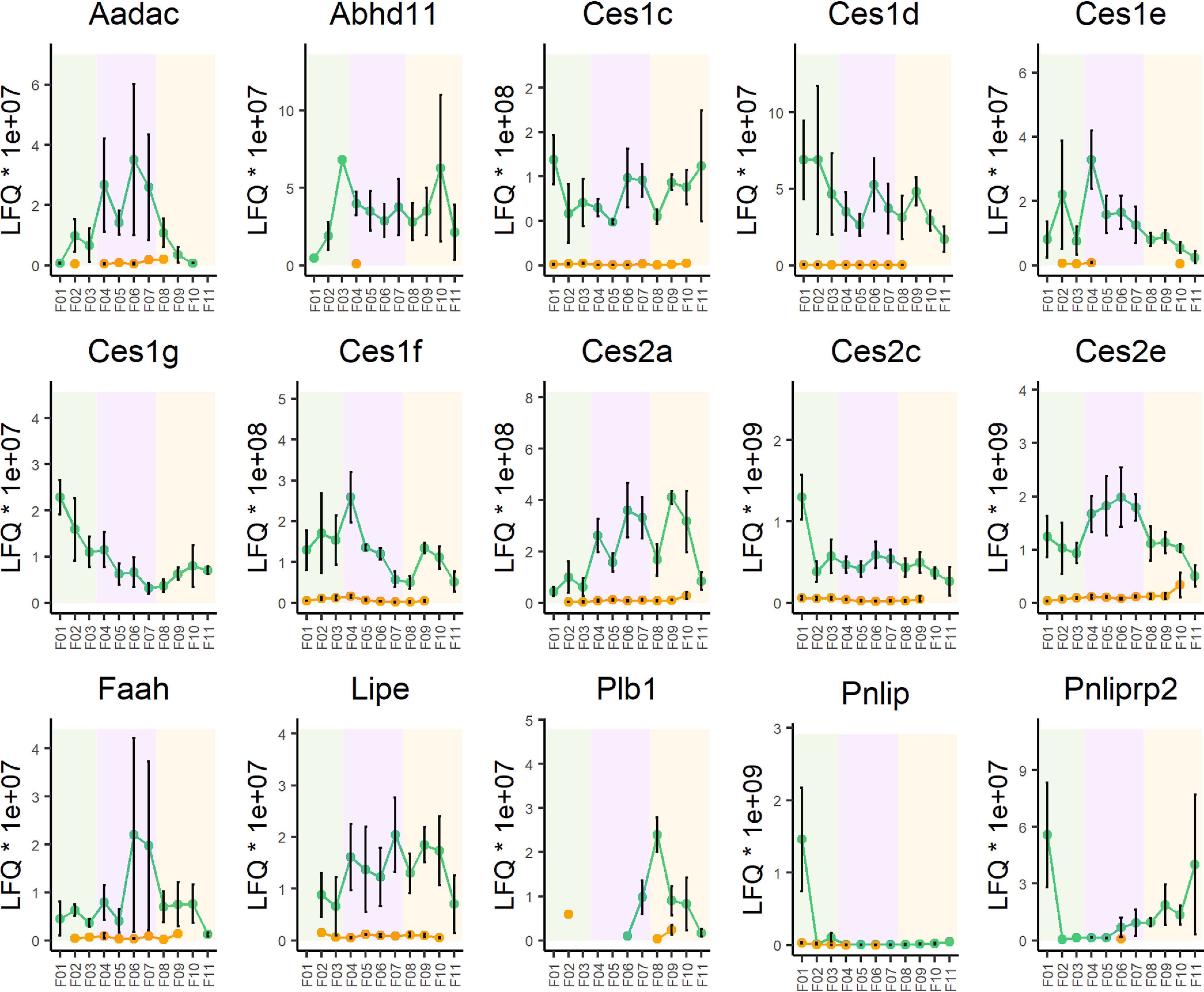
**Longitudinal activity profiles of enriched lipid hydrolases in the small intestine** (labeled with gene names). Plots show the LFQ abundance of enriched lipases in probed (*green* dots) and nonprobed samples (*orange* dots) in 11 fractions of the small intestine (*green* background: duodenum; *purple* background: jejunum; *orange* background: ileum). Fraction numbers increase the further distal the fraction was taken (Fig. 1*A*).

Activity-based enrichment profiles of the secreted luminal lipases PL and PL-RP2 showed an expected high initial activity which rapidly declines along the duodenum (Fig. 4, *Pnlip*, *Pnliprp2*). Interestingly, PL-RP2 activity increased again in the distal part of the ileum which can be explained by the reported expression of the enzyme by goblet cells mainly present in the distal part of the small intestine (35). Although the secreted esterases quickly declined along the duodenum, most members of the Ces family showed a more constant expression. Solely Ces1g and Ces2c had a clear maximum expression in the first fractions of the small intestine, with Ces2c declining more rapidly (Fig. 4). In contrast, Ces1e and Ces1f had a pronounced activity spike at the very beginning of the jejunum (fraction 4, Fig. 4) whereas Ces2e was enriched throughout the jejunal fractions. The latter enzyme was also the most abundant jejunal lipase based on LFQ (Fig. 3*E*). Two other enzymes with reported lipolytic activity were enriched exclusively in the jejunum. The activity profile of Phospholipase B-like 1 (PLBD1, Q8VCI0, Plbd1) (36) showed a very low enriched abundance throughout the small intestine (supplemental Fig. S6). Enrichment only reached significance in jejunum aided by low abundance levels or missing values in the nonprobed control. In contrast, AADAC (37) increased along the first half of the small intestine, peaking in the middle of the jejunum, to continuously decline afterward (Fig. 4). Although the abundance in the highly active fractions was only around 5 times higher as found for PLBD1, the relative activity-based enrichment compared with nonprobed control was around 40-fold over the entire section and greater than 50-fold in the most active fraction (fraction 6, Fig. 4). Interestingly, HSL (*Lipe*) and FAAH appeared to have their maximum activity in the second half of the jejunum (fractions 6 and 7, Fig. 4). The activity profile of FAAH peaked in the jejunum and gradually declined into the ileum, whereas HSL and ABHD11 showed a more even distribution. Some hydrolases were identified exclusively in the ileum but only two of them have reported lipase activity, namely PL-RP2 and membrane associated Phospholipase B1 (PLB/LIP, Q3TTY0, *Plb1*). These two enzymes have been designated as broad substrate specificity lipases (38). Although the former displayed an interesting U-shaped activity profile already described before, PLB/LIP activity sharply peaked at the beginning of the ileum to decline again as rapidly as it had risen (Fig. 4).

##### Enzyme Targets Altered between Sections

**Fig. 5.**
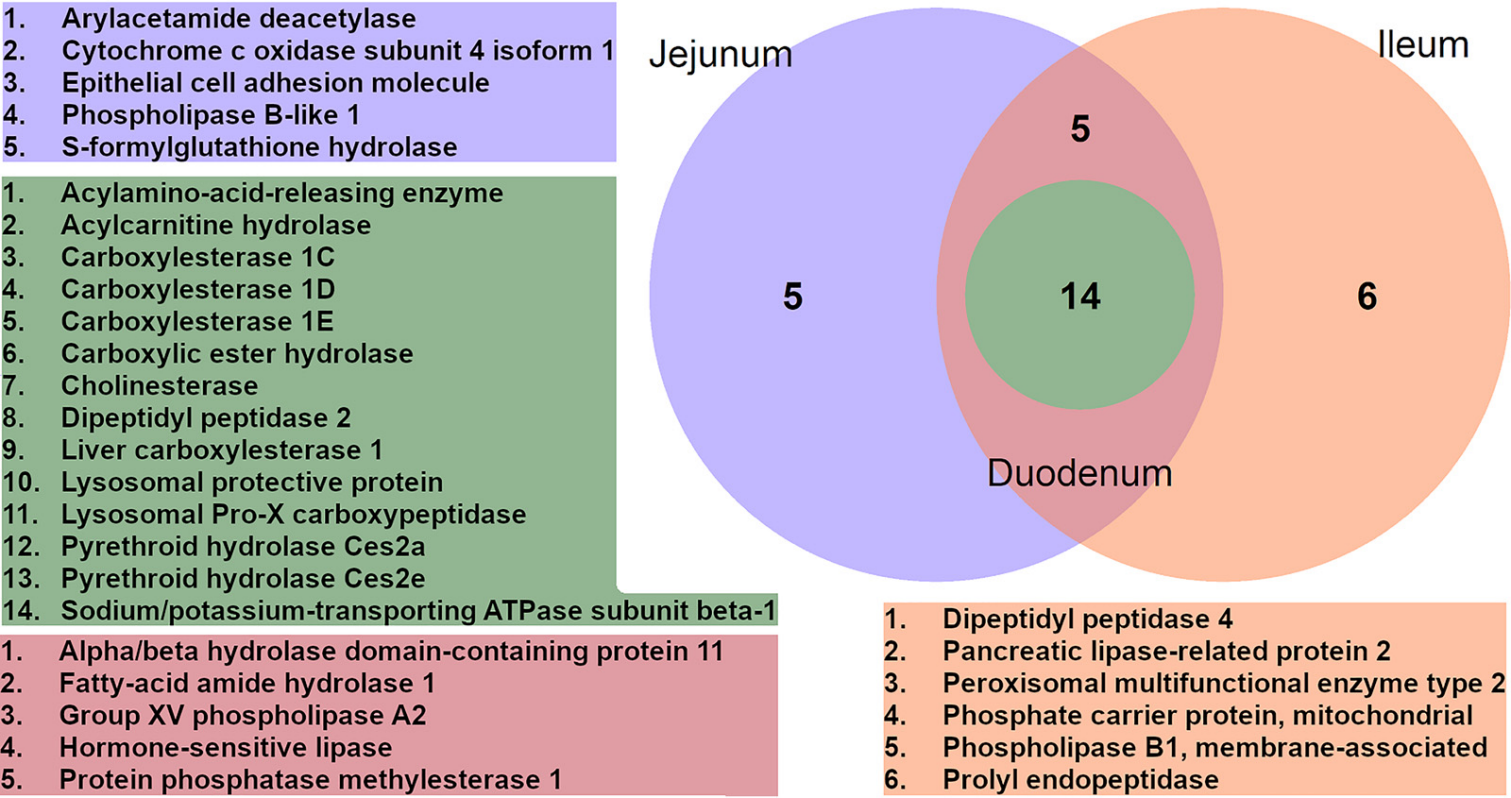
**Venn diagrams of significantly enriched enzymes in small intestinal sections.** Green: proteins enriched in all three fractions; purple: jejunum; orange: ileum; red: jejunum/ileum overlap.

Only a couple of enzymes exceeded significance thresholds for both, enrichment over nonprobed controls and altered enrichment between adjacent sections (supplemental Fig. S4, S5, supplemental Table S6, S7). To further test for alterations between different parts of the small intestine and corroborate the identified enriched lipases solely present in individual sections, the whole data set was separated into duodenum (fractions 1–3), jejunum (4–7) and ileum (8–11), filtered for two valid values per section (supplemental Table S8) and intersections were determined. As depicted in the Venn diagram (Fig. 5), most overall identified proteins was present in all three parts of the small intestine. After filtering the results for hydrolases significantly enriched per section, no enriched enzymes were found exclusively in the duodenum, even PL (p-value 0.028) did not pass significance threshold after FDR based multi-testing.

Interestingly, all identified members of the Ces family were present in all three sections. The intersection of jejunum and ileum contained HSL, ABHD11 and FAAH as well as LPLA2 and protein phosphatase methylesterase 1 (PME-1, Q8BVQ5, *Ppme1*). In both, jejunum and ileum, we identified a small set of exclusive serine hydrolases. In the jejunum these enzymes included the off-targets/nonlipases COX IV-1, S-formylglutathione hydrolase (FGH, Q9R0P3, *Esd*) and epithelial cell adhesion molecule (Ep-CAM, Q99JW5, *Epcam*), which only reached statistical significance in this intestinal section. The remaining section-specific lipases were PLBD1 and AADAC, an enzyme sharing amino acid sequence homology with HSL. Finally, the ileum-specific enzymes also contained some nonlipase targets, namely phosphate carrier protein (PTP, Q8VEM8, *Slc25a3*), peroxisomal multifunctional enzyme type 2 (MFE-2, P51660, Hsd17b4) and the two peptidases dipeptidyl peptidase 4 (DPP IV, P28843, *Dpp4*) and prolyl endopeptidase (PE, Q9QUR6, *Prep*). One of the two remaining enzymes with proposed lipase activity, PL-RP2 was studied in detail before (38, 39) and the second one, PLB/LIP, has a predicted (Uniprot) broad substrate spectrum, including TAG.

## DISCUSSION

Enterocytes are highly polarized cells that take up FFA and MAG in the form of mixed micelles from the intestinal lumen and secrete them as TAG loaded CM via the lymph to the bloodstream. Under high nutritional fat load, enterocytes can form CLD for transient lipid storage to increase uptake efficiency of dietary lipids and buffer postprandial hypertriglyceridemia (1, 8, 9). To mobilize FFA from this cytosolic TAG storage, a so far unidentified lipase must cleave these lipids for import of FFA and MAG into the ER to enable CM assembly (7). Studies using knockout mouse models of the most obvious lipase candidates (12, 13, 15, 17) failed to elucidate the mechanism of this intracellular lipid transfer. We hypothesize that this unknown hydrolase should have an activity profile similar to the distribution of CLD and CM secretion activity along the small intestine. We therefore performed an activity-based proteomics enrichment employing a hydrolase specific probe in 11 consecutive sections of the small intestine, followed by a quantitative proteomics workflow. We were able to identify 25 specifically enriched enzymes covering a wide range of known lipases, esterases and amidases.

The most prominent lipolytic enzymes ATGL, HSL and MAG lipase (MGL, O35678, *Mgll*) catalyze a lipolytic cascade from TAG to FFA and glycerol in adipose tissue. Interestingly, although we detected ATGL in selected fractions of each probed mouse but not in the nonprobed controls (supplemental Fig. S4), no significant enrichment was found even after imputing missing values. As we have reliably enriched ATGL from other cells and tissues employing the same activity-based approach, this finding indicates low expression levels and/or activity of ATGL in the small intestine. This is in line with recent results showing that ATGL/ABHD5 deletion does not affect luminal lipid absorption or CM secretion of enterocytes (14). Despite its significant enrichment in jejunum and ileum, HSL has already been ruled out as a bulk TAG hydrolase in enterocytes (13). Finally, MGL was not significantly enriched along the intestine.

Remarkably, the abundance and enrichment factors of the classical lipolytic enzymes was marginal compared with members of the Ces1 and 2 subfamilies. In line with reports based on mRNA levels (16), the Ces2 subfamily was the most abundantly expressed one throughout the small intestine. Although Ces2c showed by far the highest mRNA level in duodenum, Ces2e ranked first in the same section based on activity-based enrichment. Likewise, comparable mRNA levels of Ces2c and Ces2e levels in the jejunum and ileum translate to an activity-based enrichment of less than 30 and 50%, respectively, implying considerably higher enzymatic activity of Ces2e. Interestingly, the activity profiles of Ces family members across the small intestine varied quite strikingly. The activity of Ces1d, Ces1g and Ces2c constantly declined along the small intestine, whereas Ces1e and Ces1f showed a marked activity spike at the beginning of the jejunum (fraction 4). Ces2e, the most abundantly enriched enzyme in the whole data set, showed its highest activity in the entire jejunum (fractions 4-7). Ces2a increased up to the second last fraction of the ileum whereas Ces1c showed a relatively constant activity along the entire small intestine. Given our hypothesis that a lipase highly active in the jejunum is responsible for TAG remobilization from enterocyte CLD, we conclude that Ces1e, Ces1f and Ces2e are interesting candidates for this role. Albeit Ces family members are commonly found in the ER or microsomal fraction, which would rule out their function in CLD remobilization, D'Aquila *et al.* (40) identified Ces2 enzymes but no Ces1 family members on CLD isolated from mouse enterocytes. In combination with its activity profile corresponding well with reported CLD storage areas of the small intestine and the overall highest activity, Ces2e is a prime candidate for functional follow up studies.

Along with the Ces family the N-terminal peptidase APH was one of the most abundant hydrolases found in all sections. Based on the substrate specificity of the human ortholog (P13798) of this enzyme which preferentially cleaves acetylated peptides it is unlikely that APH has bulk TAG hydrolase activity. Specificity for acylcholines, with a clear preference for acetylcholine, was also the reason to exclude CHLE as a potential candidate despite its significant enrichment in the entire small intestine. PLB/LIP shows a very characteristic activity and is only present in the second half of the small intestine. However, its activity sharply peaks at the beginning of the ileum, which does not correlate well with reported CLD distribution. Moreover, similarity based structural prediction of this enzyme in Uniprot reveals a transmembrane domain, an unlikely feature for a CLD lipase.

Only one study so far investigated ABHD11, whose expression in adipose tissue is stimulated by high fat diet (41). Based on a BLAST search, ABHD11 shares less than 35% identity with its closest relative murine protein (PME-1), which likewise shows its highest enrichment in jejunum and ileum. Finally, AADAC has been studied in detail for its role in VLDL assembly (42), especially during hepatitis C virus infection (37). Furthermore, the yeast analog of the enzyme, Say1, was found to be active and present on CLD (43). Together with the activity profile determined in this study, which shows a clear peak in the middle of the small intestine, AADAC is also among the top candidates for playing a key role in CLD remobilization.

In conclusion, we present a map of hydrolase activities along the murine small intestine based on an activity-based proteomics approach. Aligning activity profiles with morphological characterization and previous knowledge of lipase subcellular localization allows to efficiently filter for the most prominent candidates for the mobilization of CLD in enterocytes of the jejunum. In addition, the presented activity profiles could also be employed to assign enzyme candidates for other physiological functions specific for individual sections of the small intestine.

## DATA AVAILABILITY

The MS proteomics data were deposited to the ProteomeXchange Consortium via the PRIDE (32) partner repository with the data set identifier PXD019593 and doi:10.6019/PXD019593. Annotated spectra can be viewed at MS-Viewer using the search key yxiu2pog1a.

## Supplementary Material

Supplementary Tables

Supplementary Information
