# Supplementary material for: Spatially Resolved Activity-based Proteomic Profiles of the Murine Small Intestinal Lipases
Source: Mol Cell Proteomics. 2020 Oct 6;19(12):2104–14. doi: 10.1074/mcp.RA120.002171 (PMC7710144; doi:10.1074/mcp.RA120.002171)
Supplement: Supplementary Information [file 161466_2_supp_603991_qhdrby.pdf]

Supplementary Information

Suppl. Table S1: Abbreviations of protein names. Protein abbreviations for carboxylesterases according to Holmes *et al.* [43], lowercase protein names indicate murine carboxylesterases.

Protein Name	Abbreviation	Uniprot ID	Gene name
Acylamino-acid-releasing enzyme	APH	Q8R146	*Apeh*
Acylcarnitine hydrolase	Ces2c	Q91WG0	*Ces2c*
Alpha/beta hydrolase domain-containing protein 11	ABHD11	Q8K4F5	*Abhd11*
Arylacetamide deacetylase	AADAC	Q99PG0	*Aadac*
Carboxylesterase 1C	Ces1c	P23953	*Ces1c*
Carboxylesterase 1D	Ces1d	Q8VCT4	*Ces1d*
Carboxylesterase 1E	Ces1e	Q64176	*Ces1e*
Carboxylic ester hydrolase	Ces1f	Q91WU0	*Ces1f*
Cholinesterase	CHLE	Q03311	*Bche*
Cytochrome c oxidase subunit 4 isoform 1	COX IV-1	P19783	*Cox4i1*
Dipeptidyl peptidase 2	DPP II	Q9ET22	*Dpp7*
Dipeptidyl peptidase 4;	DPP IV	P28843	*Dpp4*
Epithelial cell adhesion molecule	Ep-CAM	Q99JW5	*Epcam*
Fatty-acid amide hydrolase 1	FAAH	O08914	*Faah*
Group XV phospholipase A2	LPLA2	Q8VEB4	*Pla2g15*
Hormone-sensitive lipase	HSL	P54310	*Lipe*
Liver carboxylesterase 1	Ces1g	Q8VCC2	*Ces1g*
Pancreatic lipase-related protein 2	PL-RP2	P17892	*Pnliprp2*
Peroxisomal multifunctional enzyme type 2	MFE-2	P51660	*Hsd17b4*
Phosphate carrier protein, mitochondrial	PTP	Q8VEM8	*Slc25a3*
Phospholipase B1, membrane-associated	PLP/LIP	Q3TTY0	*Plb1*
Phospholipase B-like 1	PLBD1	Q8VCI0	*Plbd1*
Prolyl endopeptidase	PE	Q9QUR6	*Prep*
Protein phosphatase methylesterase 1	PME-1	Q8BVQ5	*Ppme1*
Pyrethroid hydrolase Ces2a	Ces2a	Q8QZR3	*Ces2a*
Pyrethroid hydrolase Ces2e	Ces2e	Q8BK48	*Ces2e*
S-formylglutathione hydrolase	FGH	Q9R0P3	*Esd*
Sodium/potassium-transporting ATPase subunit beta-1	ATP1B1	P14094	*Atp1b1*

For the following tables see SupplementaryTables.xlsx:

Suppl. Table S2: Significantly enriched enzymes in the small intestine

Suppl. Table S3: Significantly enriched enzymes in the duodenum

Suppl. Table S4: Significantly enriched enzymes in the jejunum

Suppl. Table S5: Significantly enriched enzymes in the ileum

Suppl. Table S6: Significantly altered enzymes duodenum vs. jejunum

Suppl. Table S7: Significantly altered enzymes jejunum vs. ileum

Suppl. Table S8: Proteins filtered for two valid values per section

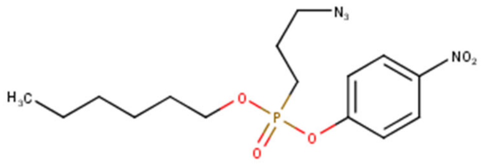

Suppl. Fig. S1: Chemical structure of the employed activity based probe (C6).

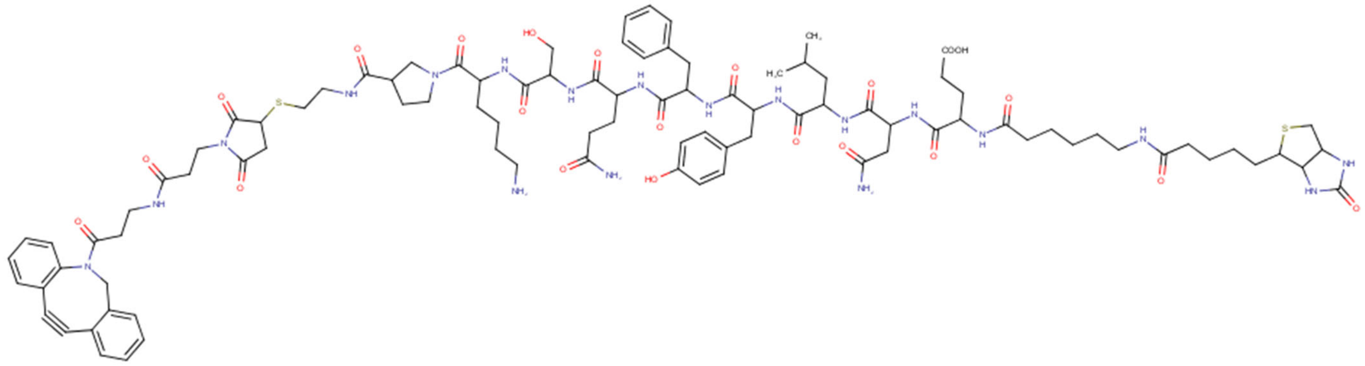

Suppl. Fig. S2: Chemical Structure of DBCO-TEV-Biotin strain promoted click linker (PiChem, Austria)

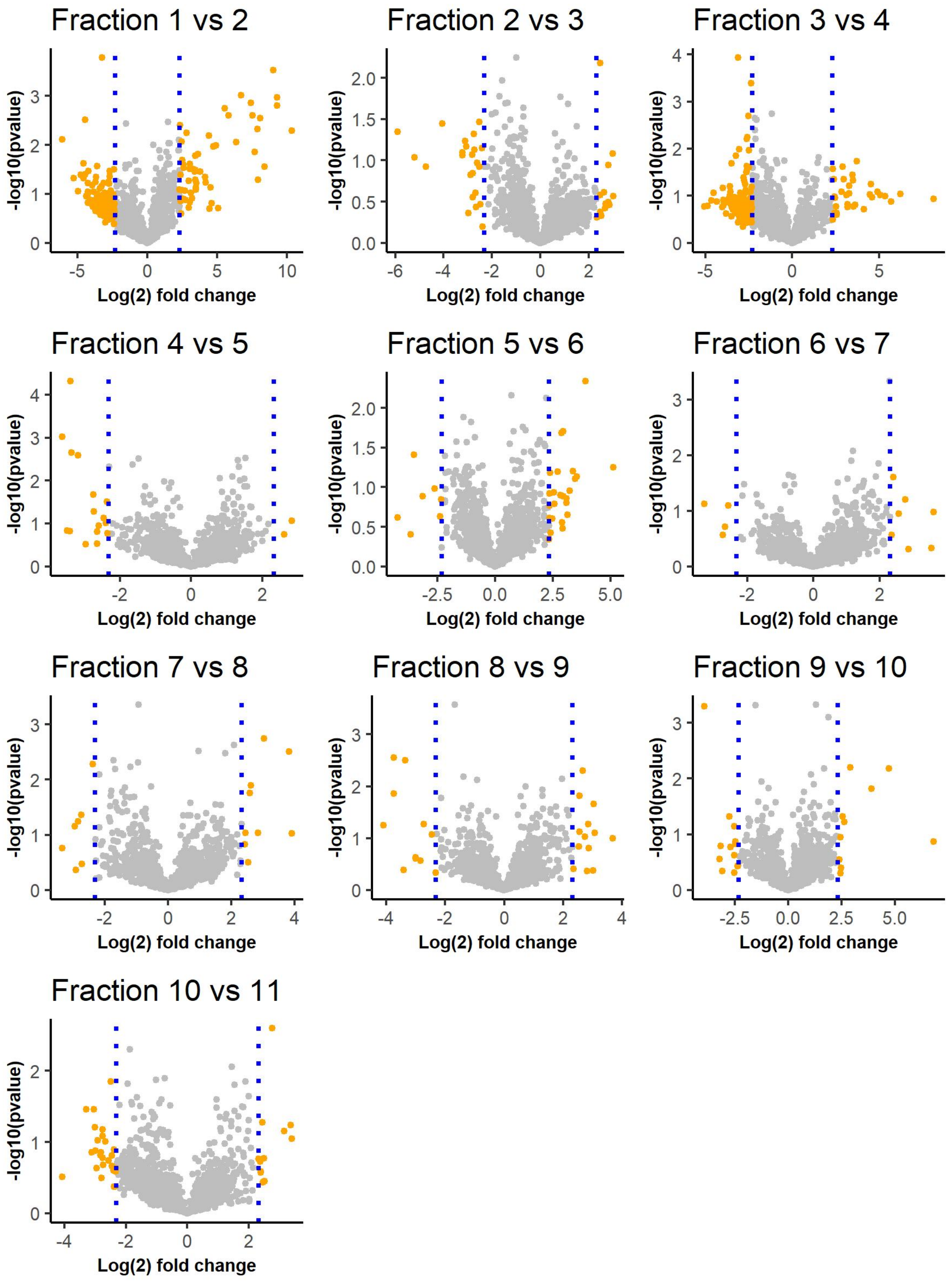

Suppl. Fig. S3: Enrichment changes between adjacent fractions. Fractions of probed animals were tested pairwise along the intestine with multi-testing correction (q < 0.05). Enrichment of no protein was found to be significantly altered between two fractions. Yellow dots show > 5-fold changed, grey dots show remaining proteins.

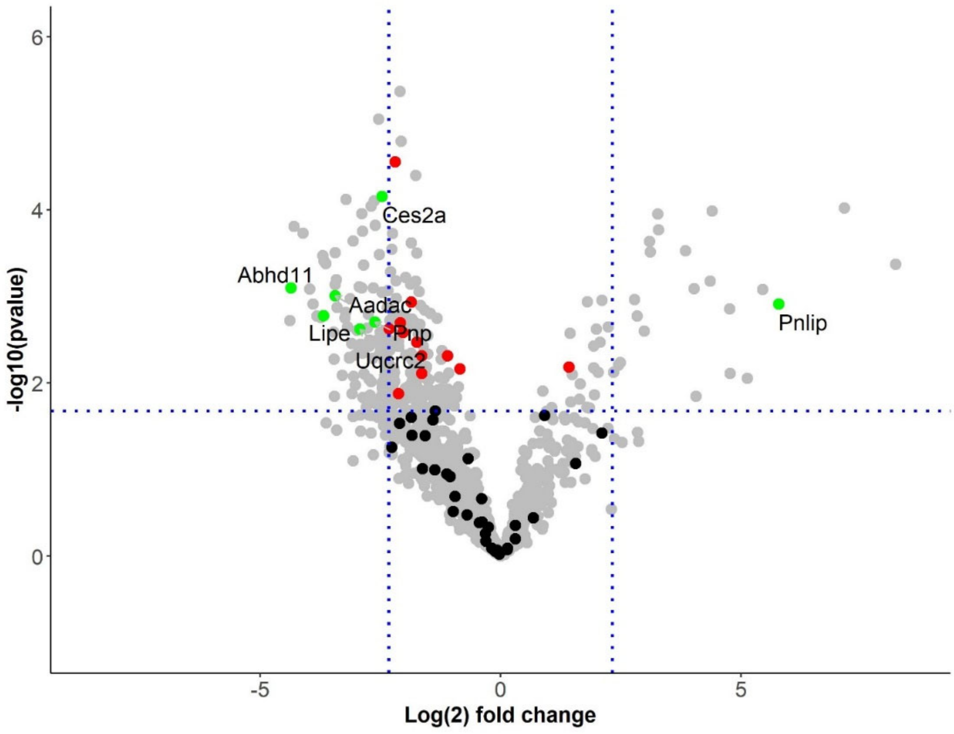

Suppl. Fig. S4: Specifically enriched enzymes found to be significantly upregulated in duodenum (right) or jejunum (left). Enriched lipases not fulfilling any significance threshold are depicted in black, those fulfilling q-value < 0.05 are depicted in red and lipases fulfilling q-value < 0.05 and fold-change > 5 are depicted in red. Background consists of all proteins identified (grey).

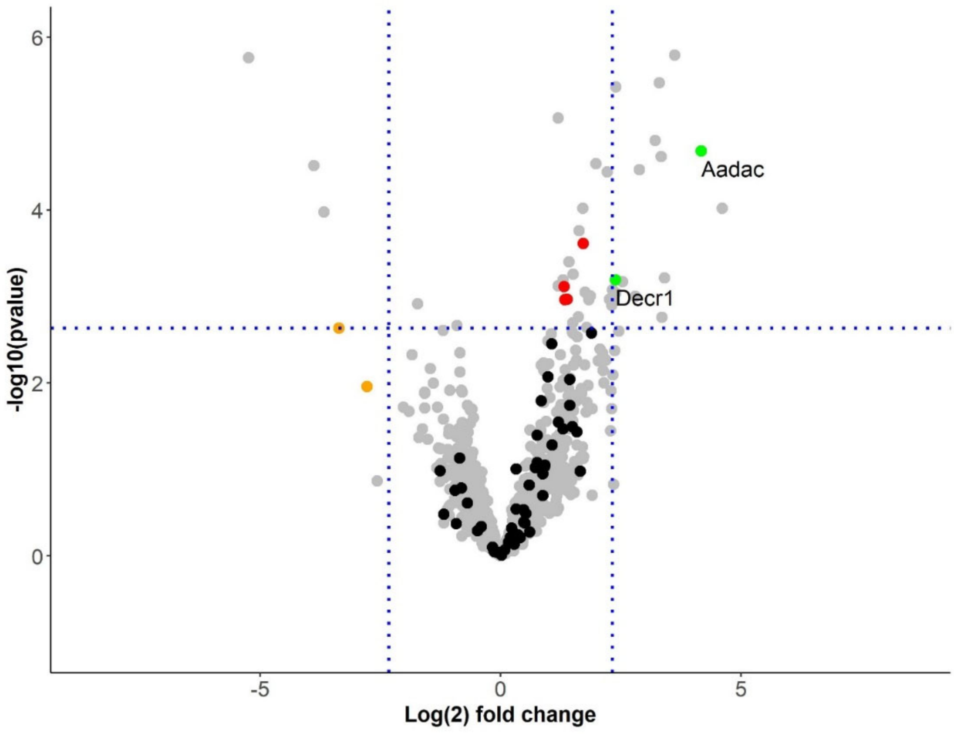

Suppl. Fig. S5: Specifically enriched enzymes found to be significantly upregulated in jejunum (right) or ileum (left). Enriched lipases not fulfilling any significance threshold are depicted in black, those

fulfilling q-value < 0.05 are depicted in red, yellow dots only fulfill fold-change > 5 and lipases fulfilling q-value < 0.05 and fold-change > 5 are depicted in red. Background consists of all proteins identified (grey).

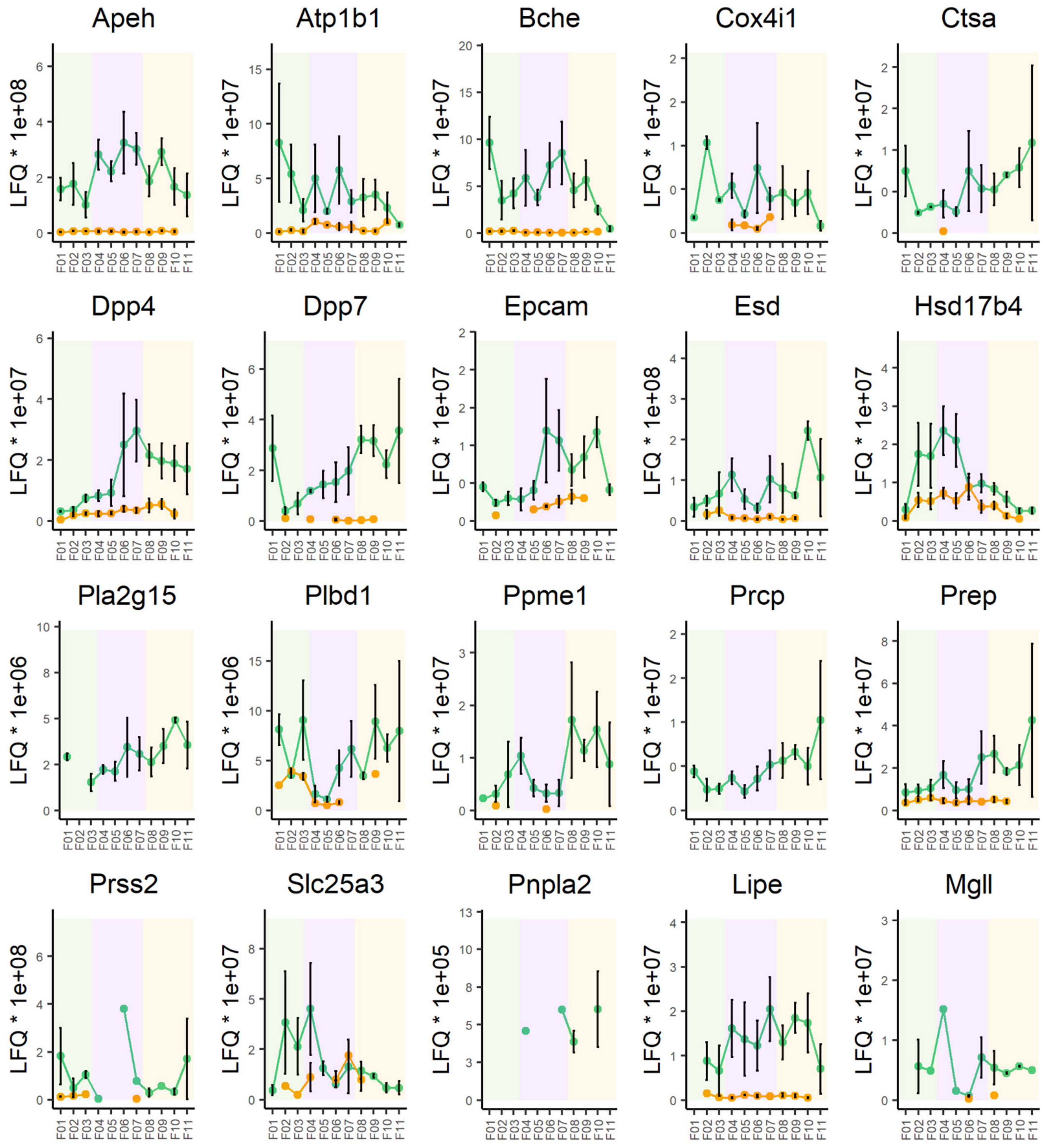

Suppl. Fig. S6: Activity profiles of peptidases and short chain esterases not considered as CLD lipase candidates and activity profiles of members of the classical lipase cascade from adipose tissue. Plots show the LFQ abundance of enriched lipases in probed (green dots) and non-probed samples (orange dots) in 11 fractions of the small intestine (green background: duodenum; purple background:

jejunum; orange background: ileum. Fraction numbers increase the further distal the fraction was taken (Fig. 1A).
